# Predictive Factors of Augmented Reality–Based Clinical Task Performance Among Novice Users: Cross-Sectional Quantitative Study

**DOI:** 10.2196/81236

**Published:** 2026-04-10

**Authors:** Amogh J Vellore, Shovan Bhatia, Michael R Kann, Nicolás M Kass, Regan M Shanahan, Jacquelyn Jardini, Jayne Miner, Sohail R Daulat, Griffin Hurt, Rishi Basdeo, Nicole Don, Jacob T Biehl, Edward G Andrews

**Affiliations:** 1 Department of Neurological Surgery, University of Pittsburgh Medical Center, 200 Lothrop St, STE B-400, Pittsburgh, PA, 15213, United States, 1 412-647-3685; 2 Department of Orthopaedic Surgery, University of Pittsburgh Medical Center, Pittsburgh, PA, United States; 3 Department of Plastic Surgery, University of Pittsburgh Medical Center, Pittsburgh, PA, United States; 4 Department of Computer Science, School of Computing and Information, University of Pittsburgh, Pittsburgh, PA, United States; 5 Department of Mechanical Engineering, Carnegie Mellon University, Pittsburgh, PA, United States

**Keywords:** mixed reality, augmented reality, virtual reality, mental rotation task, visuospatial ability, medical education, video games

## Abstract

**Background:**

Augmented reality (AR) can provide risk-free training for medical trainees, yet little is known about which learner characteristics facilitate adoption or inform training design.

**Objective:**

We aimed to identify which learner characteristics predict AR performance in novices. We hypothesized that higher visuospatial ability and greater video game experience would be associated with faster completion times and fewer errors.

**Methods:**

In this cross-sectional study, 21 undergraduate, graduate, and medical students (median age 22, IQR 21-24 years) without previous AR experience were recruited between June and December 2024. Participants completed a technology experience survey, the mental rotation task (MRT) for visuospatial ability, a standardized 7-task AR protocol mimicking clinical use on the Microsoft HoloLens 2 (hologram manipulation, orbit tracing, anatomical plane visualization, and hologram-to-object registration), and the National Aeronautics and Space Administration Task Load Index for cognitive load assessment. Outcome measures included completion time, slips (unintentional errors), and tracing quality.

**Results:**

All analyses used a significance of α=.05. MRT scores did not predict baseline performance time (Pearson *r*=0.15, 95% CI −0.32 to 0.55; *P*=.54) or error rates (*r*=0.18, 95% CI −0.27 to 0.57; *P*=.43). Participants with extensive video game experience (>5 hours/week) made fewer slips (unpaired *t* test; mean difference −2.62 slips, 95% CI −5.19 to −0.04; *P*=.047), without faster completion times (Mann-Whitney test; median difference −22 seconds, 95% CI −7.00 to 57.00; *P*=.24). Video game experience did not predict baseline performance time (Pearson *r*=−0.35, 95% CI −0.69 to 0.13; *P*=.14). Significant learning effects emerged in unadjusted analyses: completion times decreased on attempts 2 and 3 compared with attempt 1 (mixed-effects analysis: mean difference 28.75 seconds, 95% CI 12.98-44.52; *P*<.001; 28.00 seconds, 95% CI 10.75-45.25; *P*=.002, respectively) with fewer slips (Friedman test: *χ*^2^_2_=17.8; *P*<.001; Dunn post hoc: *P*=.008 and *P*<.001, respectively). Orbit tracing (Wilcoxon test: median difference −5 seconds; *P*=.004) and virtual landmark placement times improved (Friedman test: *χ*^2^_3_=14.6; *P*=.002; Dunn post hoc; *P*=.009 and *P*=.02), but physical landmark placement did not. Covariate-adjusted models revealed no significant trial-by-covariate interactions.

**Conclusions:**

Visuospatial ability does not predict clinically relevant AR performance, while extensive video game experience was associated with fewer errors. Despite previous studies emphasizing inherent learner characteristics in laparoscopy and endoscopy, covariate-adjusted models showed that AR learning curves were not significantly modified by MRT or video game experience. These findings suggest that early AR performance improvements among novice users are primarily driven by learning rather than visuospatial ability, supporting training approaches that emphasize structured practice, although the modest sample size limits detection of smaller effects.

## Introduction

The rise in augmented reality (AR) and virtual reality (VR) technology has greatly impacted a range of industries, including education, entertainment, and medicine [1,2]. AR enables the supplementation of real-world visibility with digital information, which can be shown through projections onto head-mounted displays (HMD) on headsets, smart glasses, or tablet-based displays. Within medicine, AR and VR applications continue to grow. While outcomes research remains preliminary given AR’s relative infancy, studies have found that AR subjectively increases surgeon confidence in delineating tumor margins [3]. This observation was validated by a multicenter randomized controlled trial (n=113), which observed that AR-guided robotic prostatectomies were associated with a significant decrease in subsequent positive surgical margins, a key prognostic indicator for patient survival [4]. Other measured improvements have included decreased fluoroscopy time needed to navigate difficult tissue structures [5,6]. This technology has also expanded patient education [7,8] by helping patients gain a deeper understanding of their bodies and diseases while also demonstrably decreasing procedural anxiety [8,9] and improving satisfaction [8].

As applications of AR and VR continue to expand across specialties, these technologies hold tremendous potential as risk-free training modalities, allowing medical students and resident physicians to practice procedures without jeopardizing patient safety [10]. Recent literature has shown that AR can help resident physicians learn to identify aneurysms in surgical videos [11], support medical student and resident education as a reliable and predictive simulation-based medical education modality [12-14], and minimize mental workload while simultaneously improving learning capacity [15,16].

Despite this promise, there are still some important factors to consider. Although previous studies indicate that AR can increase mental resource availability [15], enhance working memory capacity [16], and facilitate long-term information storage [16], it may also serve as a distraction for some learners [17]. Research has shown broad educational benefits, from early childhood learning in preschool [18] to secondary education [19] and postgraduate medical education [13]. However, the extent of AR integration in medical education remains varied [20].

Within medical education specifically, previous studies have indicated mixed learning outcomes. For example, AR can be beneficial for anatomy learning compared to virtual dissection tables, but not when compared to the conventional atlas method [21]. Similarly, other studies have found no difference in learning among stereoscopic 3D AR models, monoscopic 3D desktop models, or conventional atlas learning [17]. Further complicating its role, evidence suggests that individuals who have lower spatial ability, as measured by mental rotation tasks (MRTs), may benefit more from AR than their peers with higher MRT scores [21,22]. These findings indicate that the mixed effects of AR within medical education may be explained by individual differences in spatial ability.

Despite the importance of spatial ability across industries, including STEM [23-25] (science, technology, engineering, and math) and medicine [26-29], and the growing adoption of AR within medicine [30], there is still a critical gap in our understanding of how novice AR users learn to use the technology. Previous experiences, such as video game experience, have been shown to play a role in spatial ability [31] as well as in medically relevant tasks [32,33]. More recently, studies have demonstrated that video game experience is a strong predictor of baseline skills in gastrointestinal endoscopy learners [34] and of baseline performance in nonmedical VR tasks [35].

However, it remains unclear which learner characteristics (eg, visuospatial ability and previous video game experience) support the efficient adoption of AR in clinical applications and whether short, targeted exposure is sufficient for novice users to reach proficiency. This study addresses this gap by quantifying novice performance and short-term learning on a neurosurgical AR navigation task and examining how these outcomes relate to individual differences in mental rotation ability and video game experience. We hypothesize that individuals with higher visuospatial ability and, specifically, more video game experience will complete AR-based neurosurgical navigation tasks more quickly and with fewer errors. These results may indicate whether specific learner characteristics confer an advantage in AR or whether novice performance in AR is primarily influenced by learning.

## Methods

### Research Design

This study used a cross-sectional framework in which participants were recruited using convenience sampling to complete a pretest demographics survey and an assessment of visuospatial ability, followed by a series of standardized AR tasks and a posttest National Aeronautics and Space Administration Task Load Index (NASA-TLX) survey to assess subjective mental load. This paper was prepared in accordance with the Journal Article Reporting Standards [36].

### Inclusion and Exclusion Criteria

Participants comprised undergraduate, graduate, and medical students at the University of Pittsburgh between June 2024 and December 2024. Participants who had previous experience using AR were excluded.

### Ethical Considerations

**Figure 1.**
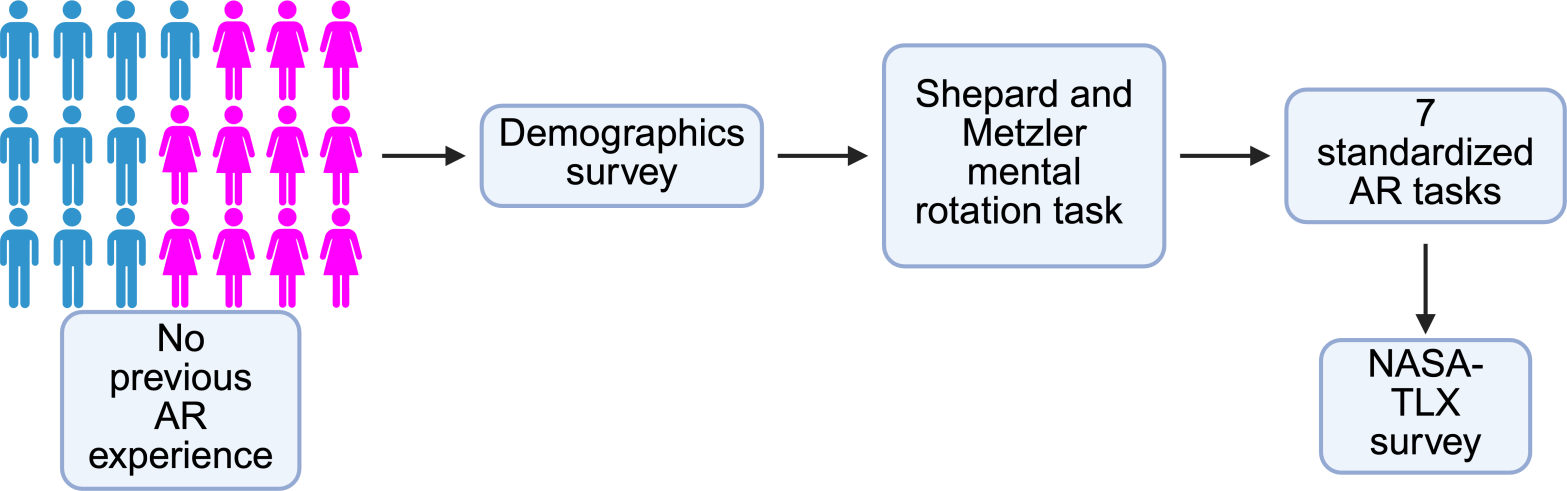
Study design. In total, 23 participants were recruited for this study between June 2024 and December 2024. Two participants did not successfully complete all tasks and were excluded from the analysis, resulting in a final cohort of 21 participants. There were 11 female (pink) and 10 male (blue) participants with no previous experience with augmented reality (AR). The demographics survey collected information such as experience with video games, comfort with new technology, and educational background. All participants then performed a series of mental rotation tasks before completing 7 standardized AR tasks. Following completion of the tasks, participants were given a posttest National Aeronautics and Space Administration Task Load Index (NASA-TLX) survey to assess workload.

Participants gave their informed consent for participation in the study, for their performance to be recorded for analysis, and for any secondary analyses without additional consent. Participants were not compensated. The authors confirm that there are no images or identifiable features within this manuscript. All participant information was deidentified, and study data were stored in an encrypted location. This study received institutional review board approval from the University of Pittsburgh (STUDY22040182). The study workflow is shown in Figure 1.

### Surveys

Two pretest tasks were administered. The first task was a survey that collected demographic and experience information such as age, sex, level of education, experience with video games, comfort with new technology, and experience with surgical devices. The second pretest task was the MRT, a standardized paper-and-pencil measure of 3D spatial visualization derived from the mental rotation paradigm by Shepard and Metzler [37]. The MRT requires participants to decide whether comparison figures are rotated versions or mirror images of a target 3D object, providing a robust index of individual differences in mental rotation ability. Classic psychometric work has shown that the MRT has high internal consistency as indicated by the Kuder-Richardson Formula 20 (Kuder-Richardson Formula 20=0.88), which estimates how consistently dichotomously scored items measure the same underlying construct. Classic psychometric work has also demonstrated that the MRT has high test-retest reliability (*r*=0.83) [38], and subsequent reviews describe it as one of the most commonly used and well-validated measures of spatial ability [39,40]. Moreover, mental rotation tests such as the MRT are routinely incorporated into spatial ability batteries and reliably predict performance in applied visuospatial tasks (eg, engineering design, navigation, and surgical endoscopy) [29,41,42]. Because our experimental tasks required participants to infer 3D relationships from 2D displays and mentally transform object orientations, we selected the MRT as the primary measure of visuospatial ability.

This task was composed of 2 sets of 12 problems. Participants were allotted 3 minutes to complete each set of questions. During this task, participants were given a warning when their remaining time reached 2 minutes, 1 minute, 30 seconds, and 10 seconds. Following AR testing, participants were given the NASA-TLX survey, a clinically validated metric for measuring mental load [43].

### Experimental Procedure

This study conducted AR-based tasks using SurgicalAR (version 1.6.1; Medivis Inc) software on Microsoft HoloLens 2. SurgicalAR is a surgical guidance system that volumetrically renders Digital Imaging and Communications in Medicine data and projects it onto an HMD, allowing for direct registration to patients. Participants were shown a generic, deidentified computerized tomography angiogram of the head. For tasks that required a stylus or pointer, a stylus tracked by the SurgicalAR system was used.

Participants were given 7 different AR-based tasks that were deliberately selected to resemble the clinical workflow steps that a neurosurgeon would perform in the operating room. Specifically, tasks 1 to 3 mimicked basic hologram interactions that may be performed while visualizing key structures or planning an operative approach. Tasks 4 to 7 were designed to follow a standard hologram-to-object registration in which 4 corresponding points were placed on the hologram and the physical object. Then, a 3D transformation was computed using the method by Horn [44] to complete the registration.

**Figure 2.**
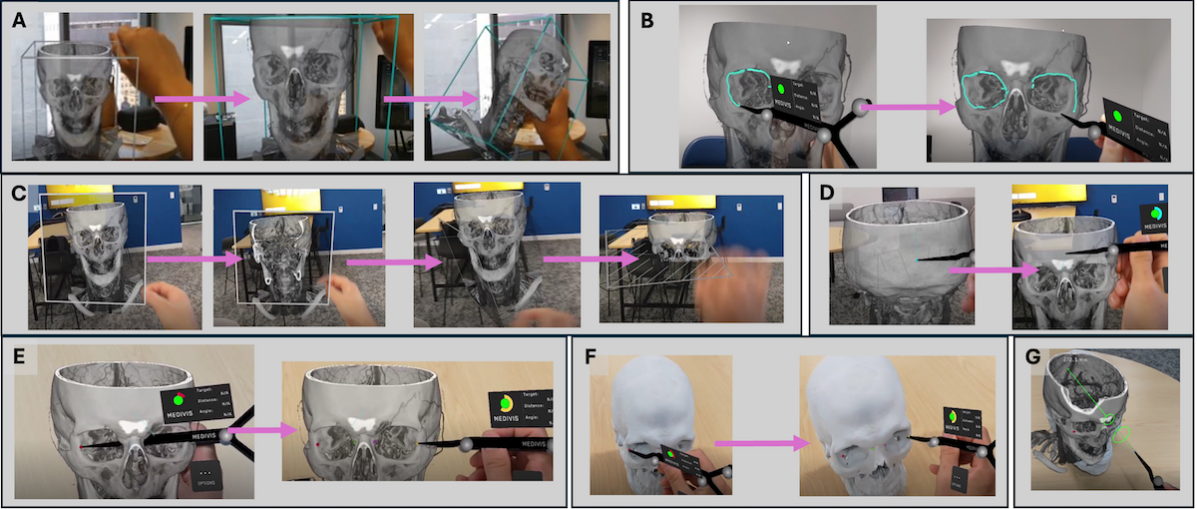
Series of augmented reality tasks that participants were required to complete. (A) Baseline performance: resizing and rotating a hologram of a human skull model; (B) orbit tracing: outlining the orbital rims on the hologram; (C) plane visualization: viewing coronal, sagittal, and axial planes of the hologram; (D) anterior-posterior trajectory point: placing virtual trajectory markers on the hologram; (E) virtual landmark placement: placing 4 virtual landmarks on the hologram; (F) physical landmark placement: placing 4 physical landmarks on the 3D-printed human skull model; and (G) trajectory alignment: performing trajectory alignment.

Before participants began using AR, the study moderator demonstrated the task using the HoloLens 2 while participants viewed the task through the SurgicalAR system cart monitor, which was positioned near the moderator. Then, the moderator gave and adjusted the headset on each participant and instructed them on basic gesture interactions. All tasks were performed on and recorded using the HoloLens 2. Videos were analyzed for performance using predefined metrics, as defined below. A description of each task is provided below, and representations of the tasks can be seen in Figure 2.

- Task 1 (Figure 2A): participants resized and rotated a hologram of a human skull model. This task required participants to unanchor the hologram, detaching it from its fixed position and allowing free movement. They then needed to make the hologram larger (zoom in) and smaller (zoom out) and rotate the hologram 360°. Finally, participants reanchored the hologram, locking it back into its original orientation and size. This task was repeated 3 times. Task performance was measured by time taken to complete and by number of slips. Slips were defined as unintentional errors or mistakes [45,46].
- Task 2 (Figure 2B): participants outlined the orbits (eye sockets) of the hologram. Participants were instructed to perform the orbit tracing in one continuous motion for each orbit, without retracting the areas they had already outlined. Performance was measured by a qualitative analysis of orbit tracing quality.
- Task 3 (Figure 2C): participants moved a cut-plane tool fully through the hologram of computerized tomography angiogram of the head in 3 directions—coronal, sagittal, and axial. They were instructed to perform the task while keeping their body facing the front of the hologram. Performance was measured by the number of slips, defined as instances in which a person intends to do one action but unintentionally does something else [45,46].
- Task 4 (Figure 2D): participants placed 2 virtual trajectory landmarks. The first point was placed midline on the lambdoid suture, and the second point was placed midline on the frontal bone. Performance was measured by the time required to successfully place the posterior point and anterior point.
- Task 5 (Figure 2E): participants placed 4 virtual landmark points on the bilateral lateral and medial parts of the hologram’s orbit. They began with the lateral left orbit and worked from left to right, finishing with the lateral right orbit. Performance was measured by the time to place each virtual landmark point.
- Task 6 (Figure 2F): participants placed 4 physical landmark points on a 3D-printed skull model, matched to the same locations as the virtual landmarks. They began with the lateral left orbit and worked from left to right, finishing with the lateral right orbit. Performance was measured by the time to place each physical landmark point.
- Task 7 (Figure 2G): participants registered the holographic computerized tomography projection onto the physical skull and then activated the trajectory alignment tool. To accomplish this, participants used the stylus to make the anterior-posterior trajectory turn green, indicating successful alignment. Performance was measured by time taken to align trajectory.

### Statistics

Descriptive statistics were used to summarize demographic variables and baseline characteristics. Group comparisons were performed using independent samples 2-tailed *t* tests for continuous variables and Pearson *χ*^2^ tests for categorical variables, where appropriate. To assess learning effects, mixed-effects models with Tukey multiple comparisons were used for completion times. Friedman tests with Dunn post hoc comparisons were used for error counts and landmark placement times, and Wilcoxon signed-rank tests were used for paired comparisons. The overall effect of trial on performance was evaluated using a 1-way repeated-measures ANOVA with Greenhouse-Geisser correction. To evaluate whether learning effects were modified by MRT scores or video game experience, a covariate-adjusted repeated-measures general linear model was used. Linear regression was used to evaluate the predictive relationship between MRT scores and baseline task performance. Participants were stratified based on video game experience (>5 hours/week vs ≤5 hours/week) to assess group differences in task outcomes. Significance was set at *α*=.05 for all comparisons. All statistical analyses were conducted using GraphPad Prism (version 10.0.0; GraphPad Software Inc).

## Results

### Overview

In total, 23 participants with no previous experience with AR were recruited for this observational study. Of these, participants 3 and 42 (8.69%) did not successfully complete all tasks and were excluded from the analysis, resulting in a final cohort of 21 (91.3%) participants. Within the final cohort, there were 11 (52.4%) female participants, and the median age was 22 (IQR 21-24) years. There were 15 (71.4%) participants who were undergraduate students. In total, 13 (61.9%) participants spent between 0 to 5 hours per week playing video games, and 10 (47.6%) participants spent between 0 to 10 hours per week interacting with a touch screen device or computer. Furthermore, 13 (61.9%) participants were completely comfortable with new technology. Specific demographic information and comfort with new technology are presented in Table 1. Results of the NASA-TLX are presented in Table 2.

**Table 1.** Participant demographics (N=21).

Variable	Value
Sex, n (%)	
Female	11 (52.4)
Male	10 (47.6)
Age (years), median (range; IQR)	22 (19-25; 21-24)
Level of training, n (%)	
Undergraduate student	15 (71.4)
Medical student	5 (23.8)
Master’s student	1 (4.76)
Time spent playing video games per week (hours), median (range; IQR)	5 (0-55; 1.5-21)
Weekly video games use (hours), n (%)	
0-5	13 (61.9)
6-10	1 (4.76)
11-15	2 (9.52)
16-20	0 (0)
≥21	5 (23.8)
Time spent interacting with touch screen device or computer (hours), median (range; IQR)	15 (0-63; 5-40)
Weekly touch screen devices or computer use (hours), n (%)	
0-10	10 (47.6)
11-20	1 (4.76)
21-30	3 (14.3)
31-40	3 (14.3)
≥41	4 (19.0)
Comfort with new technology (scale 1-5), n (%)	
Totally comfortable (5)	13 (61.9)
Very comfortable (4)	4 (19.0)
More or less comfortable (3)	3 (14.3)
Not very comfortable (2)	1 (4.76)
Not comfortable at all (1)	0 (0)
Experience with other forms of surgical guidance, n (%)	
Endoscopy^a^	2 (9.52)
DaVinci^b^	1 (4.76)
Microsurgery	0 (0)

a Average experience with endoscopy was 13 (SD 4.49) hours.

b Total experience with DaVinci was 6 hours.

**Table 2.** National Aeronautics and Space Administration Task Load Index scores.

Category	Value, median (range; IQR)
Mental demand	50 (0-80; 25-67.5)
Physical demand	15 (0-70; 5-30)
Temporal demand	35 (0-75; 10-50)
Performance (lower is better)	50 (20-85; 37.5-67.5)
Effort	50 (0-90; 30-67.5)
Frustration	30 (0-85; 12.5-60)

### Overall Performance on MRT

**Figure 3.**
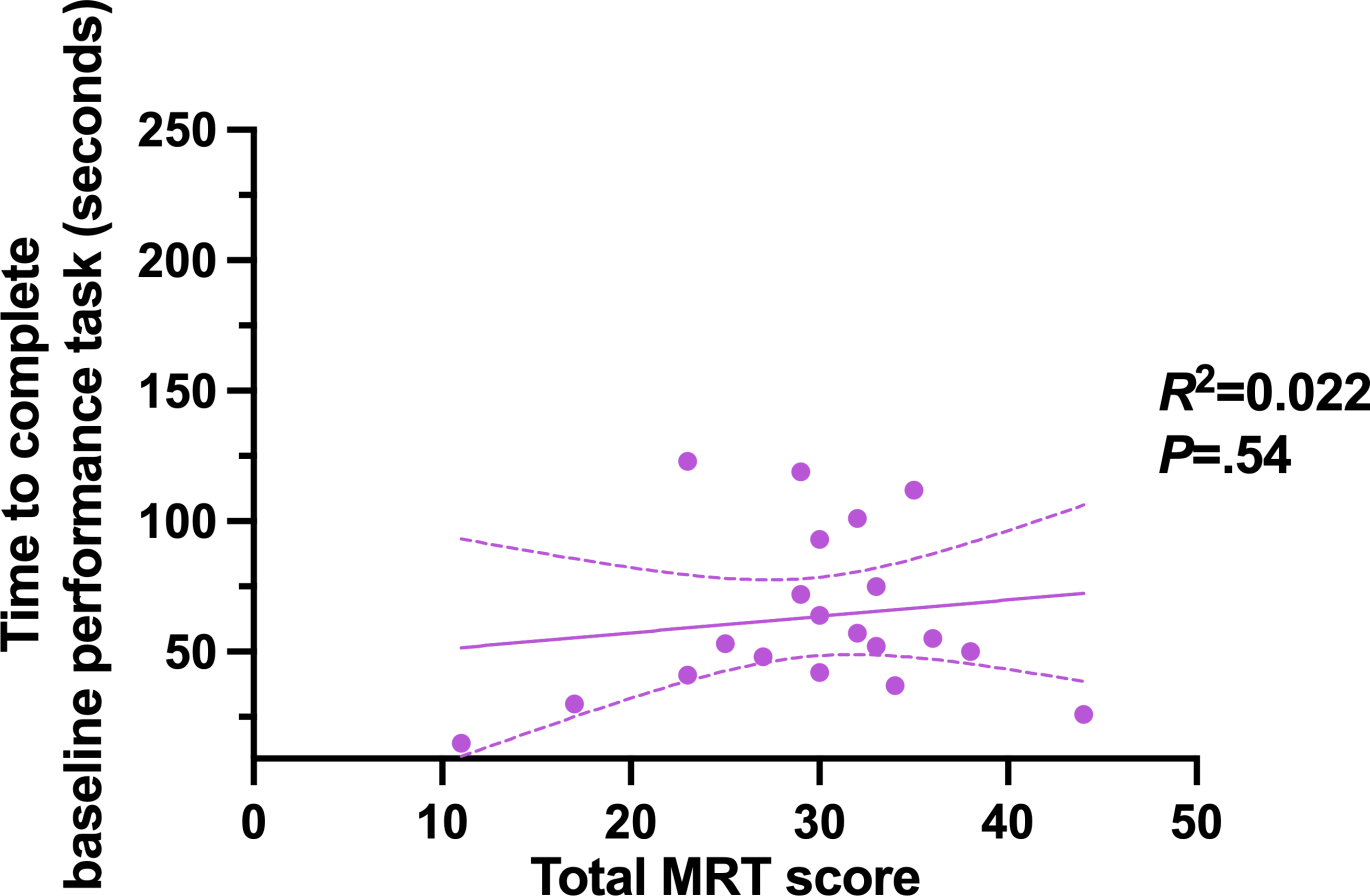
Association between visuospatial ability, measured by the mental rotation task (MRT), and baseline task completion time. Each dot represents an individual participant’s MRT score and corresponding completion time. The solid line indicates the linear regression fit, and the dashed lines represent the 95% CI of the regression. MRT score does not predict the ability to complete the baseline performance task (*R*^2^=0.022; *P*=.54).

Visuospatial ability, as measured by the MRT, did not predict the time taken to complete the baseline performance task (Pearson *r*=0.15, 95% CI −0.32 to 0.55; *R*^2^=0.022; *P*=.54; Figure 3). Similarly, MRT scores did not predict error rates on the baseline performance task (*r*=0.18, 95% CI −0.27 to 0.57; *R*^2^=0.034; *P*=.43). There were no statistically significant differences in baseline performance time (*P*=.65) or number of slips (*P*=.62) between individuals with MRT scores ≥30 and those with scores <30.

### Video Game Performance

Participants were split into 2 groups based on video game experience (group with “extensive” experience of >5 hours/week and group with “minimal” experience of ≤5 hours/week) for analysis. This distribution was determined empirically to yield approximately equal participants per group (8 and 13, respectively). Participants with extensive video game experience did not demonstrate faster completion times compared to those with minimal video game experience (Mann-Whitney test; median difference −22 seconds, 95% CI −7.00 to 57.00; *P*=.24).

However, individuals who had extensive video game experience made fewer slips on average than those who had minimal video game experience (mean 4.00, SD 2.27 slips, 95% CI 2.10-5.90 vs mean 6.61, SD 3.36 slips, 95% CI 4.59-8.64; unpaired *t* test; mean difference −2.62 slips, 95% CI −5.19 to −0.04; *P*=.047).

### Learning

**Figure 4.**
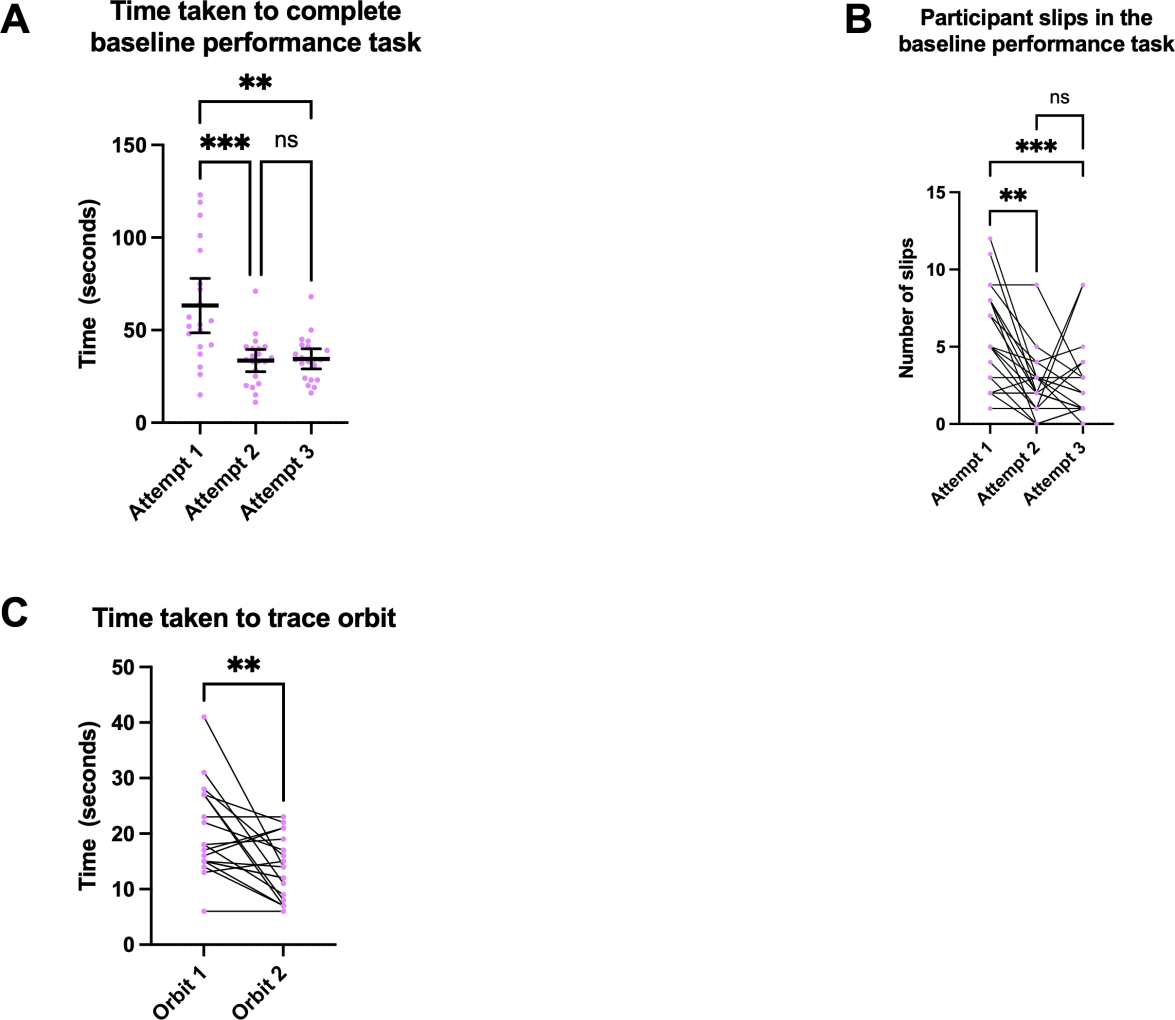
Learning effects observed across various augmented reality tasks. (A) Time taken to complete baseline performance task across attempts. Each dot represents an individual participant, and the horizontal lines indicate the mean with 95% CIs. Participants performed the baseline performance task in a significantly shorter time between attempts 1 and 2 [***P<.001] and attempts 1 and 3 [**P=.002]. The comparison between attempts 2 and 3 was not significant [ns; *P*>.99]*.* (B) Number of slips in the baseline performance task across attempts. Each dot represents an individual participant, and the connecting lines track each participant’s performance across attempts. Participants improved in the accuracy of completing the baseline performance task, as demonstrated by fewer slips between attempts 1 and 2 [**P=.008] and attempts 1 and 3 [***P<.001]. The comparison between attempts 2 and 3 was not significant [ns; *P*>.99]*.* (C) Time taken to trace orbits. Each dot represents an individual participant, and the connecting lines track each participant’s performance between orbits. Participants significantly improved the time to trace the orbits on the second attempt [**P=.004].

Participants learned to perform the baseline performance task (task 1) in a significantly shorter time between attempts 1 and 2 (mean 63.3, SD 31.4 seconds, 95% CI 48.5-78.0 vs mean 33.6, SD 13.1 seconds, 95% CI 27.6-39.6; *P*<.001) and attempts 1 and 3 (mean 63.3, SD 31.4 seconds, 95% CI 48.5-78.0 vs mean 34.5, SD 12.0 seconds, 95% CI 29.0-40.0; *P*=.002), but not between attempts 2 and 3 (mean 33.6, SD 13.1 seconds, 95% CI 27.6-39.6 vs mean 34.5, SD 12.0 seconds, 95% CI 29.0-40.0; *P*>.99; Figure 4A).

A 1-way repeated-measures ANOVA indicated that there was a significant effect of trial time, consistent with improved performance across trials (Greenhouse-Geisser *F*_1.135,22.691_=11.890; *P*=.002). However, a covariate-adjusted repeated-measures general linear model that included weekly gaming hours and MRT score indicated that the trial effect was not significant (Greenhouse-Geisser *F*_1.112,20.021_=0.050; *P*=.85), and there was no evidence that trial-related changes depended on either covariate (trial×gaming hours: *P*=.62; trial×MRT: *P*=.34).

In addition to faster task completion times between attempts 1 and 2 and 1 and 3, participants also performed the task more accurately. There was a decrease in the number of slips between attempts 1 and 2 (mean 5.62, SD 3.2 slips, 95% CI 4.2-7.1 vs mean 2.48, SD 2.02 slips, 95% CI 1.6-3.4; *P*=.008) and attempts 1 and 3 (mean 5.62, SD 3.2 slips, 95% CI 4.2-7.1 vs mean 2.57, SD 2.52 slips, 95% CI 1.4-3.7; *P*<.001; Figure 4B). Furthermore, participants completed the orbit tracing more quickly between the first and second orbit (mean 20.4, SD 8.15 seconds, 95% CI 16.5-24.4 vs mean 13.9, SD 5.52 seconds, 95% CI 11.31-16.5; *P*=.004) without a change in quality of orbit tracing (*P*=.77; Figure 4C).

Additionally, participants required less time to place virtual landmark points 2 and 3 (mean 7.2, SD 4.82 seconds, 95% CI 5.0-9.4 vs mean 4.2, SD 1.81 seconds, 95% CI 3.4-5.0; *P*=.009) and points 2 and 4 (mean 7.2, SD 4.82 seconds, 95% CI 5.0-9.4 vs mean 4.0, SD 1.82 seconds, 95% CI 3.2-4.8; *P*=.02). There was no learning effect observed for placing physical landmarks (Friedman test; Dunn post hoc: all pairwise *P*≥.64). There was no significant difference in the learning curve between participants with MRT scores ≥30 and those with scores <30 (*P*=.87). Furthermore, there was no difference in learning curves between participants with extensive video game experience and those with minimal video game experience (*P*=.81).

### Predictive Variables

MRT performance did not predict baseline performance, as measured by task 1 (*P*=.54; Figure 3). Additionally, video game experience was not a predictor of baseline performance (Pearson *r*=−0.35, 95% CI −0.69 to 0.13; *R*^2^=.12; *P*=.14); however, it did predict the number of slips (*P*=.046).

## Discussion

### Principal Findings

As AR technology continues to improve and integrate within health care and other industries, it becomes increasingly important to understand which factors contribute to technological proficiency among novice AR users. By identifying these factors, product designers can address the scarcity of implementation models that is hindering the widespread adoption of AR and VR in clinical settings [30] and develop programs to help guide novice users through more complex AR-based interactions, thereby proactively addressing areas of difficulty, minimizing the user learning curve, and increasing user adoptability. To address this growing need, our study aimed to identify predictors of performance in novice AR users. Our findings suggest that visuospatial ability does not predict AR task completion time, though extensive video game experience was associated with greater accuracy. Despite this result, neither visuospatial ability nor video game experience corresponded with an improved learning curve.

### Predictive Variables of Performance Gains

Existing literature has placed a strong emphasis on visuospatial ability as a predictor of performance in various clinical settings, including ultrasound [27], laparoscopic [28], and endoscopic procedures [29], as well as in nonclinical settings [47,48] and learning [26]. Given that factors such as depth perception and stereovision undoubtedly contribute to an individual’s visuospatial ability [49], our study used one of the most popular validated ways of evaluating spatial ability, the MRT [50,51]. In our study, we found no relationship between MRT scores and baseline performance. This suggests that AR proficiency may be influenced by more nuanced visual processing skills that are not captured by the MRT.

Höhler et al [49] and Martin-Gomez et al [52] have suggested that depth perception and stereoacuity affect individuals’ ability to estimate distances of objects in AR. Given the importance of interacting with virtual elements in AR, estimating the depth and position of these objects may play a larger role than previously thought and could account for the visual processing skills that are not captured by the MRT.

The observed result that increased video game experience was correlated with increased accuracy in AR tasks may be explained by the beneficial effect of gaming on spatial cognition. Work by Bavelier and Green [53] indicates that specifically action video game play enhances spatial cognition; however, other literature has indicated that these cognitive improvements are not unique to only action games [54]. This indicates that the relationship between video game experience and accuracy in AR may be due to the cognitive benefits of extensively playing video games, regardless of genre.

The literature indicates that video game experience may be a positive predictor of performance in surgical tasks with respect to errors and time [55-57]. Our findings suggest that this relationship may extend to AR-based applications with respect to errors; however, more research is needed to evaluate its effect on performance time.

### Learning How to Use AR

One of the reasons AR can be challenging for novice users is the variability in the learning process [58]. However, as with other skills, increased AR exposure is associated with improved performance. Our unadjusted analyses demonstrated a rapid learning effect, with the most pronounced gains occurring during early task exposure. This suggests that novice AR users may rapidly familiarize themselves with the AR environment. However, covariate-adjusted models did not indicate that these improvements differed significantly based on user characteristics.

Users who initially performed tasks more slowly demonstrated the greatest improvement. Tasks requiring less depth perception showed more rapid learning, while those emphasizing higher depth perception and precision, such as the virtual landmark placement (task 5), improved more gradually. Notably, physical landmark placement (task 6) did not show a learning effect, possibly because participants could rely on tactile feedback from touching the skull with the stylus.

Given that covariate-adjusted models showed no significant influence of MRT or video game experience on learning, these findings suggest that inherent user characteristics, such as spatial ability, do not impact early AR learning capacity in novice users. However, given our modest sample size (N=21), the nonsignificant covariate terms and interactions should be interpreted cautiously.

### Importance of Depth Perception With AR

There is a possibility that depth perception and stereoacuity play a larger role in novice AR performance due to inherent technological limitations of the HMD. The AR device used in this study, the Microsoft HoloLens 2, uses a traditional fixed plane optical display. Research with the HoloLens has supported that visual rendering factors such as shadows [59] and lighting conditions [60] may impact the depth perception of users. Additionally, binocular disparity and the occlusion of an object are other important cues for depth perception [61].

If a user attempts to interact with a virtual object in AR, they may experience an occlusion error, in which the object appears translucent despite the user’s hand not being at the appropriate distance to interact with it. Uehira and Suzuki [61] identified that this depth perception error was highly varied between individuals, particularly at short distances where the difference in binocular disparity is especially pronounced. Most of the tasks in our study were performed at short distances, mimicking clinical interactions with AR. Our study did not quantify the distances of the virtual objects, nor did we measure how many times users missed targets due to misjudgment of depth. Given that interaction with virtual objects is a fundamental component of AR use, it is likely that individuals who have stronger depth perception abilities may outperform those with weaker depth perception [49].

Concurrently, these findings provide new evidence that traditional measures of visuospatial ability do not reliably predict novice AR performance, while unmeasured factors, including depth perception, may contribute more than previously thought. The early performance gains observed in unadjusted analyses suggest that novice AR proficiency can be rapidly developed, a result supported by short-format training within urology [14]. Importantly, these learning effects, combined with the scarcity of existing implementation models [30], suggest that successful AR adoption may benefit from short, targeted training programs that guide all novice users to a competency threshold rather than prioritizing users based on traits such as visuospatial ability or video game experience. Furthermore, this emphasizes that predictive measures of novice performance should be interpreted in the context of this rapid rate of improvement.

### Limitations

This study has some notable limitations. The potential sampling bias introduced by the inclusion of only undergraduate and graduate students may limit the generalizability of the findings to broader populations, such as resident and attending physicians who represent actual AR users in health care settings. Given that our sample size was 21 nonsurgeon participants, we believe that further research evaluating the learning curve within intraoperative environments is necessary before concluding that task-specific guides will reduce the learning curve.

Additionally, as the sample was modest (N=21) and the covariate-adjusted model included multiple predictors (gaming hours and MRT), this study may be underpowered to detect small-to-moderate covariate effects and trial-by-covariate interactions. Accordingly, nonsignificant covariate terms (eg, gaming hours *P*=.80; MRT *P*=.17) and interaction terms (trial×gaming hours *P*=.62; trial×MRT *P*=.34, Greenhouse-Geisser corrected) should be interpreted cautiously. Additionally, video game experience was self-reported and categorized based on hours per week. Our study found it challenging to obtain the genre of video games played and therefore did not analyze whether different categories of video games influenced performance with AR. The cognitive demand effects and the type of video games were not collected; however, these factors may have an influence on how participants perform in the tasks we evaluated in this study. Furthermore, technical limitations of the Microsoft HoloLens 2 cannot be discounted, such as ambient lighting conditions in the room during experimentation, which may have affected hologram visual quality. Finally, some outcomes, such as orbit tracing quality, were evaluated qualitatively and may be subject to observer bias.

### Conclusions

As AR technology continues to grow in adoption across different industries, there is an increased need to identify the factors that contribute to effective AR use. Our research found that extensive video game experience was correlated with decreased error frequency, while neither visuospatial ability nor video game experience predicted novice user performance time. We believe that future research should focus on how depth perception, stereoacuity, and learning play a role in novice user performance, while also evaluating the learning curve of surgeons in intraoperative environments. This area of research holds important promise and may shape how industry professionals and product developers design and train future users to adopt AR systems more effectively.

## Abbreviations

AR: augmented reality
HMD: head-mounted display
MRT: mental rotation task
NASA-TLX: National Aeronautics and Space Administration Task Load Index
STEM: science, technology, engineering, and math
VR: virtual reality
